# Thyrotoxicosis occurrence in SARS-CoV-2 infection: A case report

**DOI:** 10.1016/j.amsu.2022.103700

**Published:** 2022-04-29

**Authors:** Maria Erika Pranasakti, Nimitta Talirasa, Henda Ageng Rasena, Rosita Yunanda Purwanto, Sumadi Lukman Anwar

**Affiliations:** a Department of Internal Medicine, Rumah Sakit Nasional Diponegoro, Semarang, Indonesia; b Rumah Sakit Akademik UGM, Yogyakarta, 55291, Indonesia; c Division of Surgical Oncology - Department of Surgery, Dr Sardjito Hospital / Faculty of Medicine, Public Health, and Nursing, Universitas Gadjah Mada, Yogyakarta, 55281, Indonesia

**Keywords:** Thyrotoxicosis, COVID-19, Crisis, Emergency, Extrapulmonary manifestations, ACE2, Angiotensin-converting enzyme 2, COVID-19, Coronavirus disease 2019, ECG, Electrocardiography, SVT, supraventricular tachycardic rhythm, TMPRSS2, Transmembrane Protease Serine 2, TSH, Thyroid Stimulating Hormone

## Abstract

**Introduction:**

Coronavirus Disease 2019 (COVID-19) is predominantly manifested as respiratory distress. There are growing reports of extrapulmonary clinical manifestations of COVID-19 in addition to the respiratory symptoms. COVID-19 has been associated with the thyroid function through Angiotensin-converting enzyme 2 (ACE2), the central mechanism through Thyroid Stimulating Hormone (TSH), and direct replication of the virus.

**Case presentation:**

A 26-year-old woman presented with complaints of palpitation and abdominal pain for three days. Because the symptoms were worsening, she was brought to the emergency room. Her temperature was 37.9 °C without any symptoms of cough, coryza, sneezing, nor headache. Physical examination revealed tremor, tachycardia with 162 beats per minute (bpm), excessive sweating, hyperreflexia of patellar reflex, and no prominent lump in the neck. Electrocardiography (ECG) showed supraventricular tachycardic rhythm (SVT) and 150 J cardioversions were performed. The ECG converted to sinus rhythm, regular, with 120 bpm. Thyroid function tests showed an elevated fT4 level (>7.77 ng/dL) and low TSH level (<0.005 μIU/mL). Chest X-ray showed slight cardiomegaly without prominent abnormality in the lungs that was confirmed with thoracic computerized tomography. The result of the rapid antigen test for COVID-19 was positive and confirmed with polymerase chain reaction testing. She was then treated in the intensive isolation room with remdesivir, anti-hyperthyroid, and supportive therapy. As her condition improved, she was shifted to a non-intensive isolation room and was discharged from the hospital at day 7.

**Discussion:**

COVID-19 could present as a thyroid crisis as the initial clinical manifestation. Clinicians should be aware that presentation of thyroid dysfunction in a patient without previous endocrine disease could be due to COVID-19 infection. Early recognition, anti-hyperthyroid therapy, and following isolation procedures for COVID-19 are required in the emergency condition.

## Highlights

• Thyroid crisis could be associated with COVID-19 infection.
• Extra-respiratory manifestations of COVID-19 infection vary among patients.
• Clinicians should be aware of thyroid crisis as a COVID-19 infection manifestation.

## 1 Introduction

The Coronavirus Disease 2019 (COVID-19) caused by the recently identified Severe Acute Respiratory Syndrome Coronavirus 2 (SARS-CoV-2) virus is primarily associated with respiratory symptoms [1]. A wide range of extrapulmonary clinical manifestations have been reported both as the initial presentations and as sequalae including arrhythmia, acute coronary diseases, thrombosis, kidney failure, gastrointestinal symptoms, and neurological deficits [2]. The SARS-CoV-2 virus uses the angiotensin converting enzyme 2 (ACE2) in combination with the transmembrane protease serine 2 (TMPRSS2) to facilitate virus entry into the host cells. Both proteins are expressed in various extrapulmonary tissues [1,2].

Thyrotoxicosis is a condition associated with elevated thyroid hormone levels in the circulation [3]. The clinical manifestations vary from asymptomatic to a potentially fatal condition [3,4]. Misdiagnosis and inadequate treatment of thyrotoxicosis might lead to dangerous complications, including atrial fibrillation, muscle weakness, osteoporosis, delirium, altered mental status, cardiovascular failure, and death [3,4]. A subset of severe COVID-19 patients with cytokine storm has been associated with overwhelming inflammatory responses in the thyroid gland. Interleukin-6 (IL-6) is reported to destroy both the structure and function of the thyroid gland, although the exact mechanisms are not yet fully explained [5]. All patients with thyroid dysfunction after COVID-19 infection have been associated with pneumonia either moderate to severe [6].

Hyperthyroidism and hypothyroidism have been reported as sequalae of COVID-19 infection [2,5,6]. However, there is a paucity of data on the clinical manifestations and severity in critically ill patients with COVID-19, and a lack of guidelines in the clinical management of thyroid crisis due to COVID-19 infection [2,7]. In young patients, COVID-19 clinical manifestations are usually presented with mild and moderate respiratory symptoms [1]. We presented a case of thyrotoxicosis of a patient with COVID-19 infection. We reported our case following the SCARE guidelines [8].

## 2 Case presentation

A 26-year-old woman arrived in the emergency room with a chief complaint of palpitations since the past three days. Her complaint had worsened and at the day of presentation she also complained about having shortness of breath and abdominal pain. The woman did not have any history of severe endocrine disorder nor autoimmune diseases. Physical examination revealed profuse sweating, tachycardia with 162 beats per minute (bpm), nausea, hyperreflexia of patellar reflex, mild anxiety, and no prominent lump in the neck. Because the electrocardiography (ECG) revealed supraventricular tachycardia (SVT) rhythm she received cardioversions (150 J). The ECG converted to sinus rhythm, regular, with 120 bpm. Thyroid function tests were then performed that revealed a state of thyroid crisis with high fT4 (>7.77 ng/dL), and low TSH (<0.005 μIU/mL), while no TSH-stimulating antibody testing was available in our center. Complete blood counts showed white blood cells 8400/mm^3^, hemoglobin 11.5 g/dL, normal limits of coagulation test results, normal liver and renal function test results, and mild hypomagnesemia (1.56 mg/dL). Ultrasonography of the neck showed a non-enlarged thyroid gland with slight edema, a marked increase of vascularization, and no additional lesion was observed. Chest X-ray showed slight cardiomegaly without prominent abnormality in the lungs and it was confirmed with computerized tomography. Results from the rapid antigen test for COVID-19 was positive and confirmed with polymerase chain reaction (PCR) tests. She was then transferred to the intensive isolation room and treated with remdesivir 200 mg iv once/day on the first day, followed with 100 mg iv once/day on day 2–5. She also received thyrozol 20mg once a day, propranolol 40mg three times a day, Vitamin D 5000 units once a day orally and intravenously 1000 mg vitamin C once a day. As her condition was improved, she was shifted to non-intensive isolated room and was discharged from the hospital at day 7. She was then shifted to the non-intensive isolation room and was discharged on the day 7. She was advised to take orally thyrozol and propranolol until the next evaluation at the outpatient clinic. In the follow-up 1 week later, symptoms of hyperthyroidism were resolved and the thyroid function test results improved (normal free T4). There was complete symptom resolution in the following month with maintenance of oral thyrozol 20 mg once a day and propranolol 20 mg three times a day.

## 3 Clinical discussion

Here, we presented a case of a thyroid storm in association with COVID-19 infection. The patient was not directly suspected for COVID-19 infection until the positive swab test was reported. The association of thyroid dysfunction and COVID-19 infection has not been fully explained [2,5]. ACE2 and TMPRSS2 are two transmembrane proteins which are fundamentally required in the internalization of SARS-CoV-2 into host cells, thus contributing to the pathogenesis of COVID-19 [9]. These two proteins are expressed in numerous tissues including in the thyroid gland. The highest levels of ACE2 expression and activity were found in the small intestines, kidneys, heart, salivary glands, testicles, and thyroid, whereas lower levels were observed in the brain, skin, pituitary gland, and skeletal muscles [2,10]. Follicular cells lining the colloid lumen express ACE2 protein that could mediate SARS-CoV-2 internalization to further induce inflammation in the thyroid gland [2]. The virus internalization is mediated by integrin αvβ3 protein through its ability to bind to the Arg-Gly-Asp (RGD) and Lys-Gly-Asp (KGD) structures available both in the ACE2 protein and SARS-CoV-2 spike protein [2,11]. It is suggested that thyroid hormones also bind to the integrins in the cell membranes causing activation of cytokine genes that subsequently contribute in the thyrotoxicosis [11,12].

SARS-CoV-2 infection can also induce immune responses through activation of CD4 + and CD8 + T cells and persist during the resolution phase of COVID-19 infection [13]. In response to the infection, several cytokines and chemokines, including Interleukin (IL)-1β, Tumor Necrosis Factor (TNF) alpha, interferon (IFN) gamma, and monocyte chemoattractant protein 1 will be activated [14,15]. In patients with severe symptoms, the abundant systemic inflammation can cause thyroid dysfunction [14,15]. In addition, SARS-CoV-2 is able to divert immunotolerance causing primary thyrotoxicosis, exacerbating earlier thyroid disorder, initiating an idiopathic orf immune-mediated thyroiditis, or inducing a recurrence [16,17].

A meta-analysis involving 8 studies demonstrated that thyroid diseases were associated with poor prognosis in patients with COVID-19 (HR 2.48) [17]. Severity of systemic inflammation during COVID-19 infection, particularly IL-6 level has been associated with thyroid dysfunction [5]. COVID-19 patients with thyroid dysfunction have been associated with worse prognosis and longer hospital stay [5]. Uncontrolled of hyperthyroidism might lead to unfavorable cardiovascular outcome, including arrhythmia, myocardial infarction, and hemodynamic instability [7,10,18].

There have been reports of patients with Graves' disease following COVID-19 infection, particularly for patients with previous history of endocrine or thyroid diseases. Only a few cases showed the life-threatening period of a thyroid storm [19]. There were only two reported cases showing severe thyroid crisis that were diagnosed concurrently with COVID-19 infection [20]. The reported cases showed thyrotoxicosis in which COVID-19 infections triggered the autoimmune reaction or reactivation of previous immune related diseases [20]. The severe thyrotoxicosis leading into supraventricular tachycardia (SVT) in our case could be driven by systemic inflammation due to COVID-19 infection. Rehman et al. have systematically reviewed thyroid dysfunction among patients with COVID-19 infection and have shown that 1 out of 10 patients experienced SVT [21]. The dysfunction of electrical conduction causing SVT could cause life-threatening events that need early recognition and prompt treatment. In our case, the primary presentation of thyroid crisis and SVT at the emergency room has not been previously reported. With the evolution of natural diseases with COVID-19 infection, the spectrum of clinical presentations has expanded into extra-pulmonary manifestations including those that cause emergency conditions such as Torsade's de Pointes and complete heart block [22,23]. Clinicians should bear in mind the potential presentation of COVID-19 infection with thyroid crisis. Early recognition and adjustment of antithyroid medications could lead to the successful management.

Our report highlights the primary presentation of COVID-19 infection as a thyroid crisis. In the situation with a high level of community transmission, screening and the COVID-19 separation pathways are required to limit the transmission within the hospital [[24], [25], [26]]. Routine screening and testing might be required particularly in the emergency room and before the patient's admission as primary presentations of COVID-19 infection diverge from the more common range of conditions [24,27].

## 4 Conclusion

Thyrotoxicosis in patients with COVID-19 infection is rare. Hyperthyroid management in addition to the COVID-19 acute treatment might lead to an effective treatment. Direct virus injury and damage from inflammation reactions are suggested as the possible mechanisms of thyroid dysfunction in patients with acute COVID-19 infection through ACE-2 and TMPRESS2 proteins in the thyroid cells as well as cytokines and inflammatory mediators.

## Sources of funding

This report did not receive specific funding.

## Ethics approval

Not applicable.

## Consent for publication

Written informed consent was obtained from the patient for reporting the case and displaying the relevant images. Clinical images and related materials de-identified. A copy of the written informed consent is available for review by the Editor-in-Chief of this journal on request.

## Authors’ contributions

MEP and SLA conceptualized the report and finalized the manuscript. NT, HAR, RYP collected demographic, clinical, and follow-up data. All authors read and approved the final manuscript.

## Registration of research study

Not applicable.

## Guarantor

SLA.

## Provenance and peer review

Not commissioned, externally peer reviewed.

## Availability of data and materials

The clinical and imaging data supporting the analysis and findings of this study will be available from the corresponding author upon reasonable request.

## Declaration of competing interest

No potential competing interest has been declared from all authors.
